# Difelikefalin in Chinese patients with chronic kidney disease-associated pruritus

**DOI:** 10.1093/ckj/sfag031

**Published:** 2026-02-06

**Authors:** Li Zuo, Liangying Gan, Tianjun Guan, Weiming He, Hong Ye, Qingfeng Peng, Bin Liu, Houyong Dai, Yinggui Ba, Lanfen Xue, Murray Lowe, Milica Enoiu

**Affiliations:** Department of Nephrology, Peking University People’s Hospital, Beijing, China; Department of Nephrology, Peking University People’s Hospital, Beijing, China; Department of Nephrology, Zhongshan Hospital Xiamen University, Xiamen, China; Department of Nephrology, Jiangsu Province Hospital of Chinese Medicine, Nanjing, China; Department of Nephrology, The Second Affiliated Hospital of Nanjing Medical University, Nanjing, China; Department of Nephrology, Zhuzhou Central Hospital, Zhuzhou, China; Department of Nephrology, Wuxi People’s Hospital, Wuxi, China; Department of Nephrology, Affiliated Hospital of Nantong University, Nantong, China; Department of Nephrology, Qinghai University Affiliated Hospital, Xining, China; Department of Nephrology, Shijiazhuang People’s Hospital, Shijiazhuang, China; Clinical Development Department, CSL Vifor, Glattbrugg, Switzerland; Clinical Development Department, CSL Vifor, Glattbrugg, Switzerland

**Keywords:** China, chronic kidney disease-associated pruritus, difelikefalin, haemodialysis, quality of life

## Abstract

**Background:**

Chronic kidney disease-associated pruritus (CKD-aP) is a common and debilitating condition in patients receiving in-centre haemodialysis. This study evaluated the efficacy and safety of difelikefalin (a new, intravenous treatment for moderate-to-severe CKD-aP approved in >40 countries) in a Chinese population receiving haemodialysis.

**Methods:**

This Phase 3, multicentre, randomized placebo-controlled study included a 12-week double-blind treatment period, during which patients received either difelikefalin or placebo, followed by an optional 14-week open-label extension, in which all patients received difelikefalin. Endpoints included change from baseline in the Worst Itching Intensity Numerical Rating Scale (WI-NRS) at Week 4 (primary endpoint), the percentage of patients achieving a clinically relevant ≥3-point change in WI-NRS, patient-reported health-related quality-of-life measures, and incidence of adverse events.

**Results:**

Difelikefalin-treated patients showed significantly greater improvements in least squares mean change from baseline in WI-NRS at Week 4 of the double-blind period compared with placebo [difelikefalin minus placebo: −0.81 (95% confidence interval: −1.25, −0.37); *P* = .0003], and patients with ≥3-point improvement in WI-NRS at Week 12 (48.7% versus 33.8%, nominal *P* = .0235). Difelikefalin also improved health-related quality of life, with clinically relevant changes observed in 5-D itch and Skindex-10 in most patients at Week 4 of the open-label extension (estimated ≥62% and ≥56%, respectively). Adverse events were mostly mild or moderate in severity and generally consistent with the known safety profile of difelikefalin.

**Conclusion:**

Difelikefalin effectively reduced pruritus intensity and improved quality of life in Chinese patients with CKD-aP receiving haemodialysis, supporting its use in this population.

KEY LEARNING POINTS **What was known:**

- Chronic kidney disease-associated pruritus (CKD-aP) is prevalent amongst patients with chronic kidney disease (CKD) undergoing haemodialysis (HD) and negatively affects quality of life and clinical outcomes. Despite this, it remains under-recognized and undertreated.
- Difelikefalin (a synthetic kappa-opioid receptor agonist) has demonstrated efficacy in treating moderate-to-severe CKD-aP in patients receiving HD in Phase 3 clinical trials, and is approved in >40 countries, including the USA, the European Union countries, and Japan.
- Before this study, no data were available on the efficacy of difelikefalin for treating moderate-to-severe CKD-aP in Chinese patients receiving in-centre HD.

**This study adds:**

- In this Chinese population, difelikefalin treatment resulted in significantly greater improvement from baseline in Worst Itching Intensity Numerical Rating Scale (WI-NRS) at Week 4 versus placebo. The proportion of patients achieving a clinically meaningful ≥3-point improvement in WI-NRS was comparable to that observed in global and Japanese Phase 3 studies.
- Difelikefalin also led to sustained, clinically relevant improvements in health-related quality of life, as measured by the 5-D Itch and Skindex-10 scales.
- Safety findings over 26 weeks of treatment were generally consistent with the known safety profile of difelikefalin in other populations.

**Potential impact:**

- These findings support the use of difelikefalin as a treatment option for moderate-to-severe CKD-aP in Chinese patients receiving HD.
- This study highlights the need to raise awareness of CKD-aP and its treatment in China, as most patients were not receiving anti-pruritic medication despite over half reporting severe symptoms.

## INTRODUCTION

Chronic kidney disease-associated pruritus (CKD-aP) is experienced by >60% of patients with chronic kidney disease (CKD) undergoing haemodialysis (HD) [1]. The condition negatively affects health-related quality of life (HRQoL), contributing to depression, anxiety, sleep disturbance, and poor clinical outcomes, including increased mortality [2–5].

In China, >80% of patients undergoing HD are bothered by pruritus [2, 6], while 19% are very much/extremely bothered [2] and up to 57% of those bothered by pruritus may suffer from moderate-to-severe symptoms [6].

Despite its prevalence, CKD-aP remains under-recognized and undertreated [7–9]. Approved treatment options for CKD-associated pruritus (CKD-aP) are limited; among them, Nalfurafine is authorized for use in Japan, South Korea, and China [10, 11]. While off-label options are available, they often lack efficacy or tolerability [12–14].

Difelikefalin, a synthetic kappa-opioid receptor agonist [15] for the treatment of moderate-to-severe CKD-aP in patients receiving HD, is approved in >40 countries, including the USA, EU, and Japan [16–19]. Difelikefalin does not readily cross the blood–brain barrier, limiting undesirable central nervous system (CNS) effects [20]. The efficacy of difelikefalin was previously demonstrated in Phase 3 studies outside China: KALM-1 (USA), KALM-2 (global) [21], and MR13A9-5 (Japan) [22].

This Phase 3 study (NCT05885737; https://clinicaltrials.gov/study/NCT05885737) evaluated the efficacy and safety of intravenous difelikefalin (0.5 μg/kg) in Chinese patients with CKD on thrice-weekly HD with moderate-to-severe pruritus.

## MATERIALS AND METHODS

### Study design

This Phase 3, multicentre, placebo-controlled, randomized study was conducted at 35 sites in China and included a 12-week, double-blind, placebo-controlled treatment period followed by an optional 14-week, open-label extension (OLE).

Patients were randomized 1:1 to receive intravenous 0.5 µg/kg difelikefalin or placebo thrice-weekly post-dialysis for 12 weeks during the double-blind treatment period. In the OLE, all patients received difelikefalin. A 1-week follow-up period occurred following the final administration of investigational product.

### Patient population

Eligible patients were aged 18 to 85 years, with CKD, receiving HD thrice weekly and experiencing moderate-to-severe CKD-aP [defined as a mean of Worst Itching intensity Numeric Rating Scale (WI-NRS) scores ≥5, from WI-NRS scores recorded for ≥4 days during a 7-day run-in period]. Full inclusion and exclusion criteria are listed in the Supplementary Material.

### Ethical approval

The study was conducted in accordance with the principles of the Declaration of Helsinki, the International Conference on Harmonisation guidelines for Good Clinical Practice, and applicable local regulatory requirements. The study protocol was approved by an appropriate Independent Ethics Committee before implementation.

### Outcomes and assessments

#### Efficacy outcomes (double-blind period)

The primary efficacy outcome was the mean change from baseline at Week 4 of the double-blind period in the weekly mean of the daily 24-hour WI-NRS score, referred to here as the WI-NRS.

Secondary efficacy outcomes included percentage of patients achieving ≥3-point or ≥4-point improvements from baseline in WI-NRS at Weeks 4, 8, and 12, change from baseline in the weekly mean of the WI-NRS score for each week of the double-blind period, change from baseline in 5-D itch scale total score and Skindex-10 total score at Weeks 4, 8, and 12, and Patient Global Impression of Change (PGI-C) at Week 12.

#### Open-label extension outcomes

The 5-D itch total score and Skindex-10 total score were used to assess the change from baseline in HRQoL at each time point of the OLE. Greater than 5-point and 15-point changes in 5-D itch and Skindex-10 total scores, respectively, represented clinically relevant changes [21].

#### Safety outcomes

Adverse event (AE) incidence was recorded throughout both study periods.

### Statistical analysis

Sample size determination is detailed in the Supplementary Material.

The primary efficacy endpoint was analysed for the full analysis set (defined as patients who were randomized, received ≥1 dose of investigational product, and had a baseline WI-NRS score) using a mixed-effect model for repeated measures (MMRM) that included treatment, week, and treatment-by-week interaction as fixed effects. Baseline WI-NRS score, previous anti-pruritic medication use, and specific medical conditions were used as covariates. Full details and sensitivity analyses for the primary endpoint are described in the Supplementary Material.

Secondary efficacy endpoints were analysed using the full analysis set. Significance level was set at a two-sided α of 0.05, with no adjustment for multiplicity. For WI-NRS responder analysis (≥3- or ≥4-point improvement), missing WI-NRS scores were imputed using a multiple imputation, missing at random approach. Differences in the proportion of patients with an improvement from baseline in weekly mean WI-NRS score for each treatment group were compared using odds ratios (taking the placebo group as a reference) from a logistic regression model containing terms for treatment group, baseline WI-NRS score, previous anti-pruritic medication use, and specific medical conditions. Odds ratios were recorded alongside their two-sided 95% confidence interval (CI) and *P* value from the model.

The observed number and percentage of patients achieving ≥3- and ≥4-point improvement were recorded alongside their two-sided 95% CI.

Total scores and domains of the 5-D itch scale and Skindex-10 were analysed using the same MMRM as the primary efficacy analysis. PGI-C responses were analysed descriptively, and missing data were not imputed.

Safety data are presented descriptively.

Full details of statistical analyses for the secondary endpoints are presented in the Supplementary Material.

## RESULTS

### Subject disposition

**Figure 1:**
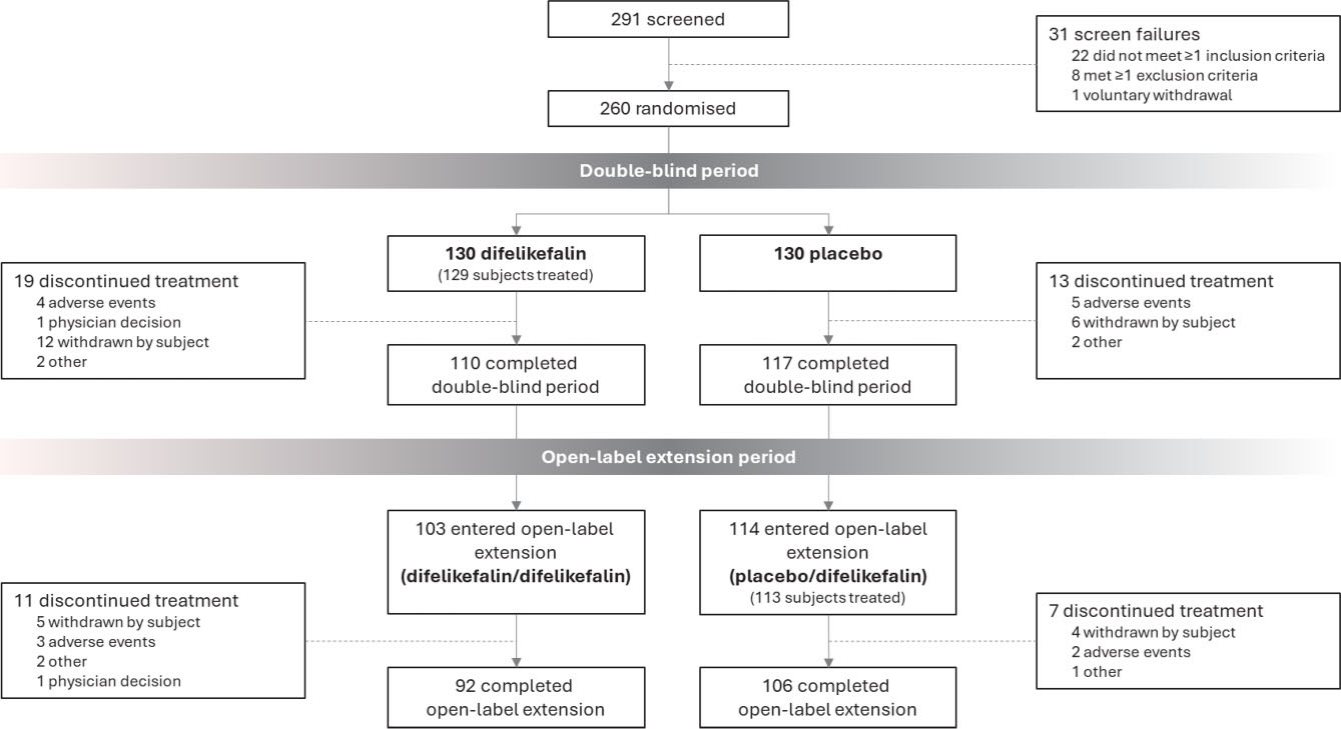
Subject disposition.

Subject disposition is shown in Fig. 1. Of 291 patients screened, 130 patients were randomized into each of the treatment arms (260 patients randomized in total), 129 of the 130 patients randomized to the difelikefalin group received study treatment (one patient withdrew before treatment), and all 130 patients randomized to the placebo group received placebo treatment (259 patients treated in total). Of these, 110 difelikefalin-treated and 117 placebo-treated patients (227 patients in total) completed the double-blind period. Following this, 103 difelikefalin-treated and 113 placebo-treated patients (216 patients in total) entered the OLE, which was completed by 92 difelikefalin/difelikefalin and 106 placebo/difelikefalin patients (198 patients in total).

### Demographics and baseline characteristics

Baseline demographics and clinical characteristics are presented in Table 1. Mean [standard deviation (SD)] age was 56.0 (12.30) years, 66.8% of patients were male, and all were Chinese with Chinese parents.

**Table 1:** Baseline demographics and clinical characteristics.

	Placebo (*n* = 130)	Difelikefalin (*n* = 129)	Total (*N* = 259)
Age at informed consent, years			
Mean (SD)	55.7 (12.38)	56.3 (12.26)	56.0 (12.30)
Median (IQR)	57.0 (47.0–66.0)	59.0 (48.0–65.0)	58.0 (47.0–65.0)
Gender, *n* (%)			
Male, %	67.7	65.9	66.8
Female, %	32.3	34.1	33.2
Prescription dry body weight (kg), mean (SD)	61.4 (10.63)	61.1 (10.94)	61.3 (10.77)
Aetiology of CKD^a^, *n* (%)			
Glomerulonephritis	42 (32.3)	32 (24.8)	74 (28.6)
Other	29 (22.3)	35 (27.1)	64 (24.7)
Hypertension	24 (18.5)	23 (17.8)	47 (18.1)
Unknown	20 (15.4)	23 (17.8)	43 (16.6)
Diabetes	17 (13.1)	20 (15.5)	37 (14.3)
Nephrotic syndrome	1 (0.8)	7 (5.4)	8 (3.1)
Cystic	0	2 (1.6)	2 (0.8)
Vasculitis	0	1 (0.8)	1 (0.4)
Hereditary	0	1 (0.8)	1 (0.4)
Interstitial nephritis	1 (0.8)	0	1 (0.4)
Urologic	1 (0.8)	0	1 (0.4)
Time since first diagnosis of CKD, years			
*n* (missing)	125 (5)	121 (8)	246 (13)
Mean (SD)	12.56 (7.977)	11.67 (7.947)	12.12 (7.958)
Median (IQR)	10.83 (6.11–16.99)	9.83 (5.93–15.98)	10.01 (6.01–16.45)
Duration of haemodialysis, years			
Mean (SD)	8.01 (5.097)	7.52 (5.630)	7.77 (5.364)
Median (IQR)	7.64 (4.31–10.33)	6.80 (2.42–11.57)	7.03 (3.54–10.83)
Time since diagnosis of ESRD (years)			
*n* (missing)	128 (2)	128 (1)	256 (3)
Mean (SD)	8.59 (5.293)	8.31 (5.893)	8.45 (5.592)
Median (IQR)	7.79 (4.85–11.10)	6.99 (3.37–12.67)	7.66 (4.16–11.69)
Duration of CKD-aP, years			
*n* (missing)	129 (1)	126 (3)	255 (4)
Mean (SD)	4.44 (3.997)	3.97 (3.645)	4.21 (3.827)
Median (IQR)	3.72 (1.60–5.06)	2.94 (1.16–5.98)	3.33 (1.33–5.18)
Specific medical conditions at baseline,^b^ *n* (%)			
Present	12 (9.2)	10 (7.8)	22 (8.5)
Absent	118 (90.8)	119 (92.2)	237 (91.5)
Weekly mean of the daily 24-hour WI-NRS score at baseline			
Mean (SD)	7.00 (1.223)	7.35 (1.291)	7.18 (1.267)
Percentage with moderate pruritus (WI-NRS ≥4 to ≤7), *n* (%)	63 (48.5)	49 (38.0)	112 (43.2)
Percentage with severe pruritus (WI-NRS ≥7), *n* (%)	67 (51.5)	80 (62.0)	147 (56.8)
Mean total 5-D itch score at baseline			
Mean (SD)	15.5 (3.29)	15.9 (3.36)	—
Mean total Skindex-10 score at baseline			
Mean (SD)	28.8 (15.16)	29.6 (15.77)	—
Anti-pruritic medication use during the week prior to randomization, *n* (%)			
Yes	15 (11.5)	14 (10.9)	29 (11.2)
No	115 (88.5)	115 (89.1)	230 (88.8)

Data are shown for the full analysis set and were available for all patients (unless missing numbers are given).

^a^Multiple responses possible.

^b^History of fall or fracture (related to fall), confusional state or mental status change or altered mental status or disorientation, gait disturbance or movement disorder.

Treatment groups were generally well-balanced with respect to the demographic and baseline characteristics. The mean (SD) WI‑NRS score was 7.35 (1.291) in the difelikefalin group and 7.00 (1.223) in the placebo group. The percentages of patients with moderate pruritus (WI-NRS ≥ 4 to ≤7) in the difelikefalin and placebo groups were 38.0% and 48.5%, respectively, and the percentages with severe pruritus (WI-NRS ≥ 7) were 62.0% and 51.5%, respectively. Most patients (89.1% and 88.5% in the difelikefalin and placebo groups, respectively) did not receive anti-pruritic treatment in the week before randomization. Glomerulonephritis was the most frequent aetiology of CKD in both treatment groups.

### Primary efficacy endpoint

**Figure 2:**
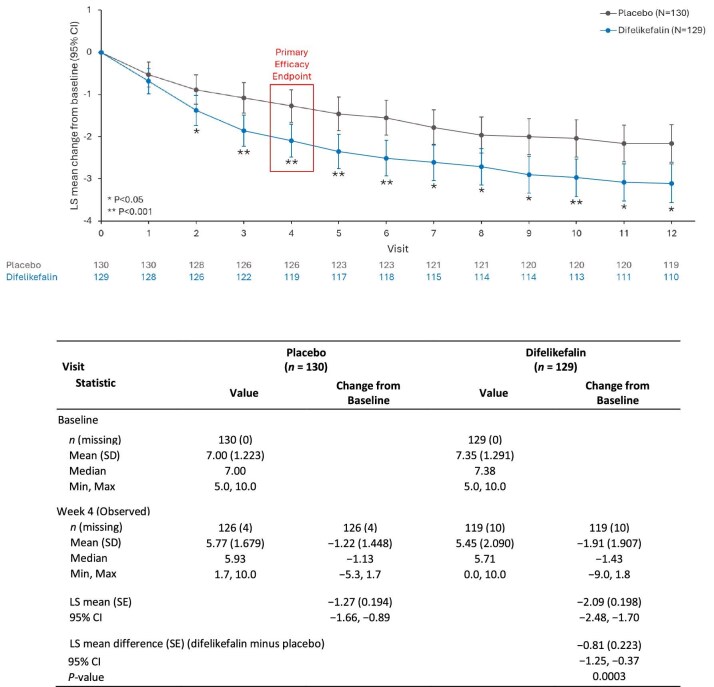
LS mean change from baseline in WI-NRS score by week during the double-blind period (no imputation). CI, confidence interval; LS, least squares; MMRM, mixed model for repeated measures; SD, standard deviation; SE, standard error; WI-NRS, Worst Itching Intensity Numerical Rating Scale. Number of patients included in the analysis at each visit are shown below the chart. Baseline WI-NRS score is calculated using all available non-missing scores collected on or before day of randomization, and previous date/time of first treatment. The MMRM includes use of previous anti-pruritic medication (yes/no), presence of specific medical conditions at baseline (yes/no), treatment, visit, and treatment-by-visit-interaction as fixed categorical effects, and baseline WI-NRS score as fixed continuous effects. Variance–covariance structure is unstructured. Repeated measures up to Week 12 are included in the model.

Difelikefalin-treated patients had a significantly greater improvement in least squares (LS) mean (95% CI) change from baseline in WI-NRS score at Week 4 of the double-blind period compared with those treated with placebo [−2.09 (−2.48, −1.70) in the difelikefalin group versus −1.27 (−1.66, −0.89) in the placebo group, LS mean difference between the groups was −0.81 (−1.25, −0.37), *P* = .0003] (Fig. 2). Sensitivity analyses of the primary endpoint (Supplementary Table 1) support the results of the primary analysis.

Comparisons of the primary endpoint data with data from studies conducted in other countries are shown in Supplementary Fig. 1.

### Secondary efficacy endpoints

#### ≥3-Point and ≥4-point reduction in WI-NRS

**Figure 3:**
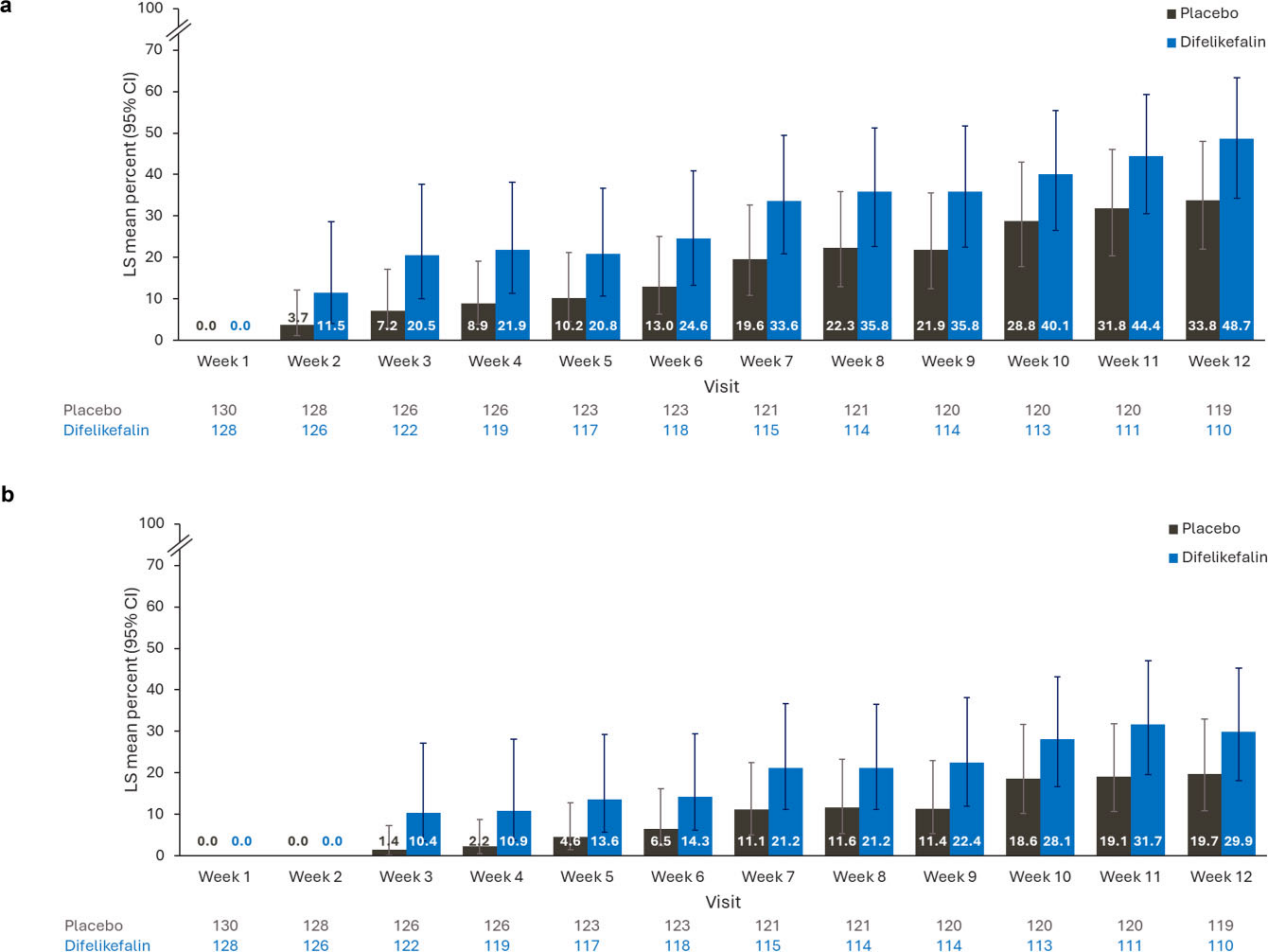
Percentage of patients with (a) ≥3-point or (b) ≥4-point improvements in WI-NRS by week during the double-blind period. MAR, missing at random; MI, multiple imputation. Number of patients included in the analysis at each visit are shown below the chart. LS mean percentage and its 95% CI are estimated using a logistic regression model with terms for treatment group, baseline WI-NRS score, use of anti-pruritic medication during the week before randomization, and the presence of specific medical conditions at baseline. Missing values are imputed using MI under MAR missing data assumption using data from across all study periods (i.e. all 12 weeks of the double-blind period). The number of patients with available data is displayed (all missing data for the logistic regression analysis are imputed).

The LS mean estimated percentages of patients with ≥3-point improvements in WI-NRS increased over time and were greater in the difelikefalin group (from 21.9% at Week 4 to 48.7% at Week 12) versus the placebo group (from 8.9% at Week 4 to 33.8% at Week 12) (Fig. 3a).

Similarly, the LS mean percentage of patients with ≥4-point improvements in WI-NRS increased over time and were greater in the difelikefalin group (from 10.9% at Week 4 to 29.9% at Week 12) versus the placebo group (from 2.2% at Week 4 to 19.7% at Week 12) (Fig. 3b).

Comparisons of the percentage of patients with ≥3-point WI-NRS improvement from baseline between this study and studies conducted in other countries are shown in Supplementary Fig. 2.

#### Changes from baseline and clinically relevant changes in 5-D itch

**Figure 4:**
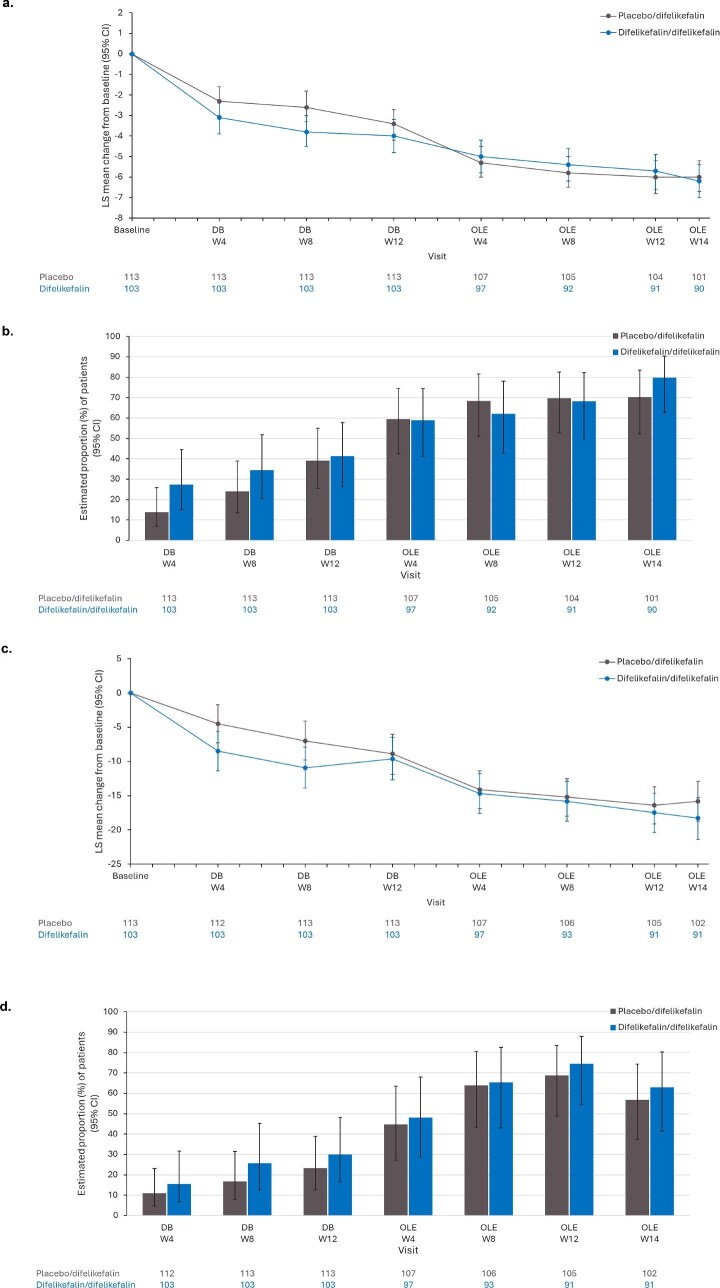
Changes from baseline and clinically relevant improvements in 5-D itch and Skindex-10 total score: (a) change from baseline in 5-D itch total score, (b) percentage of patients with a ≥5-point improvement in 5-D itch total score, (c) change from baseline in Skindex-10 total score, (d) percentage of patients with a ≥15-point improvement in Skindex-10 total score. DB, double-blind; W, week. Number of patients included in the analysis at each visit are shown below the chart. Baseline (double-blind baseline) is defined as the last available value (scheduled or unscheduled) before or on the same day as randomization or if missing as any values during Week 1 (Day ≤7). Changes from baseline (a and b): The MMRM includes previous use of anti-pruritic medication (yes/no), presence of specific medical conditions at baseline (yes/no), treatment sequence, visit, and treatment sequence-by-visit interaction as fixed categorical effects. For (a), the baseline 5-D itch score (total or domain scores) is included as a fixed continuous effect; for (b), the baseline Skindex-10 Scale score is used instead. The variance–covariance structure is unstructured. The percentage of patients with clinically relevant improvements (c and d): LS mean percentage and its 95% CI are estimated using a logistic regression model with terms for treatment sequence, use of anti-pruritic medication during the week before randomization, presence of specific medical conditions at baseline, and either baseline total 5-D Itch score (c) or baseline Skindex-10 Scale score (d) as covariates. Analysis is based on all patients who entered the OLE.

Greater improvements in 5-D itch total score were observed in the difelikefalin versus the placebo group during the double-blind period. LS mean (95% CI) change from baseline in the difelikefalin and placebo groups was −3.3 (−4.1, −2.6) versus −2.5 (−3.2, −1.8) at Week 4, −3.9 (−4.7, −3.2) versus −2.8 (−3.5, −2.0) at Week 8, and −4.3 (−5.1, −3.5) versus −3.6 (−4.4, −2.8) at Week 12 (Supplementary Table 2). Improvements continued over the OLE (Fig. 4a), with an LS mean (95% CI) change from baseline in 5-D itch total score of −6.2 (−7.0, −5.4) in the difelikefalin/difelikefalin group and −6.0 (−6.7, −‍5.2) in the placebo/difelikefalin group recorded at the end of the OLE (Fig. 4a).

The percentage of patients in the placebo/difelikefalin group with a clinically relevant ≥5-point improvement in 5-D Itch total score increased from 39.2% at double-blind Week 12 to 59.5% at open-label Week 4 (Fig. 4b). Throughout the remainder of the open-label treatment period (from Week 8 to Week 14), both groups maintained an estimated percentage of ≥62% of patients with a ≥5-point improvement (Fig. 4b). At the end of the OLE, the LS mean percentage of patients with a ≥5-point increase in the 5-D itch total score was 79.8% for the difelikefalin/‌difelikefalin group and 70.2% for the placebo/difelikefalin group.

#### Changes from baseline and clinically relevant changes in Skindex-10

Greater improvements in Skindex-10 total score were observed in the difelikefalin versus the placebo group during the double-blind period. LS mean (95% CI) change from baseline in the difelikefalin and placebo groups was −9.1 (−12.0, −6.3) versus −5.1 (−8.0, −2.3) at Week 4, −11.3 (−14.3, −8.3) versus −7.4 (−10.3, −4.5) at Week 8, and −10.5 (−‍13.6, −7.4) versus −9.5 (−12.5, −6.4) at Week 12 (Supplementary Table 3). Improvements continued over the OLE (Fig. 4c), with an LS mean (95% CI) change from baseline in Skindex-10 total score of −18.3 (−21.4, −15.3) in the difelikefalin/difelikefalin group and −15.8 (−18.7, −12.9) in the placebo/difelikefalin group at the end of the OLE (Fig. 4c).

The percentage of patients in the placebo/difelikefalin group with a clinically relevant ≥15-point improvement in Skindex-10 total score increased from 23.3% at Week 12 of the double-blind treatment period to 44.7% at Week 4 of the OLE (Fig. 4d). Throughout the remainder of the open-label treatment period (from Week 8 to Week 14), both groups maintained an estimated percentage of ≥56% of patients with a ≥15-point improvement in Skindex-10 total score (Fig. 4d). By the end of the OLE (Week 14), an LS mean of 63.0% of continuously treated patients and 56.8% of switched patients had a ≥15-point increase in Skindex-10 total score.

#### Patient global impression of change

**Figure 5:**
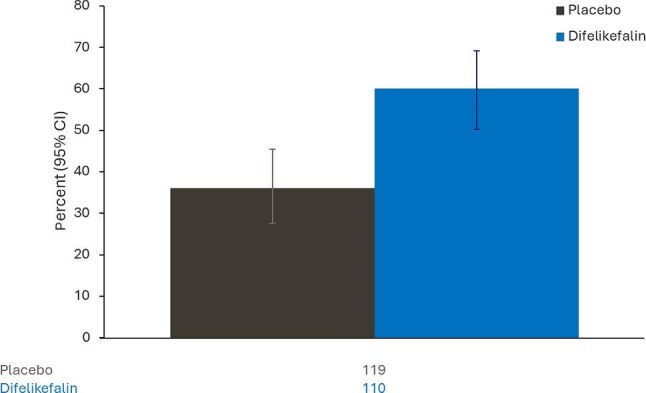
Percentage of responders in Patient Global Impression of Change at the end of Week 12 of the double-blind treatment period. Number of patients included in the analysis are shown below the chart. Responders were defined as patients who reported that their pruritus was ‘very much improved’ or ‘much improved’. CIs for the percentages are calculated as exact Clopper-Pearson CIs.

The percentage (95% CI) of responders (defined as patients whose PGI-C responses were ‘Very much improved’ or ‘Much improved’) at Week 12 of the double-blind treatment period was 60.0% (50.2, 69.2) in the difelikefalin group and 36.1% (27.5, 45.4) in the placebo group, with an odds ratio (95% CI) of 2.65 (1.49, 4.62) (Fig. 5; Supplementary Table 4).

### Safety

During the double-blind period, AEs occurred in 82.2% of patients receiving difelikefalin and 70.8% receiving placebo. In the OLE, AEs were reported in 79.6% of both the difelikefalin/difelikefalin and placebo/difelikefalin groups (Table 2). Most AEs were mild or moderate in severity (Table 2). The most common AE in both periods was hyperkalaemia [double-blind: 16.3% versus 16.9% (difelikefalin versus placebo); OLE: 19.4% and 18.6% (difelikefalin/difelikefalin versus placebo/difelikefalin)] (Supplementary Table 5). Other frequently reported AEs (≥10% in any group) were dialysis hypotension (double-blind: 14.7% versus 6.9%; OLE: 12.6% versus 14.2%) and hypotension (double-blind: 16.3% versus 6.2%; OLE: 11.7% versus 14.2%). Frequently reported AEs during the double-blind period only were muscle spasms (14.0% versus 8.5%), dizziness (13.2% versus 6.2%), and upper respiratory tract infections (10.1% versus 10.8%) (Supplementary Table 5). Treatment-related AEs occurred in 38.0% (difelikefalin) and 28.5% (placebo) during the double-blind period, and 17.5% and 23.0% during the OLE (Table 2). Hyperkalaemia was the most frequent treatment-related AE in all treatment groups (double-blind: 8.5% versus 10.0%; OLE: 10.7% versus 8.8%) (Table 2). Serious AEs occurred in 14.0% (difelikefalin) and 9.2% (placebo) during the double-blind period, and in 18.4% and 10.6% during the OLE. Treatment-related serious AEs occurred in 2.3% and 1.5% during the double-blind period and 1.0% and 0.9% during the OLE (Table 2). Study drug was interrupted due to AEs in 14.0% (difelikefalin) and 9.2% (placebo) of patients during the double-blind period, and 6.8% and 9.7% the during the OLE. Discontinuations due to AEs occurred in 3.1% and 4.6% of patients during the double-blind period, and 2.9% and 0.9% during the OLE. No AEs led to death, and none led to drug discontinuation in more than one person.

**Table 2:** Summary of adverse events.

	Double-blind treatment period		OLE period	
Adverse events *n* (%) [E]	Placebo (*n* = 130)	Difelikefalin (*n* = 129)	Placebo/difelikefalin (*n* = 113)	Difelikefalin/ difelikefalin (*n* = 103)
AEs	92 (70.8) [383]	106 (82.2) [501]	90 (79.6) [466]	82 (79.6) [407]
Mild AEs	53 (40.8)	61 (47.3)	54 (47.8)	44 (42.7)
Moderate AEs	31 (23.8)	38 (29.5)	32 (28.3)	32 (31.1)
Severe AEs	8 (6.2)	7 (5.4)	4 (3.5)	6 (5.8)
Treatment-related AEs	37 (28.5) [118]	49 (38.0) [102]	26 (23.0) [92]	18 (17.5) [38]
SAEs	12 (9.2) [20]	18 (14.0) [27]	12 (10.6) [21]	19 (18.4) [24]
Treatment-related SAEs	2 (1.5) [3]	3 (2.3) [5]	1 (0.9) [1]	1 (1.0) [1]
AEs leading to study drug interruption	12 (9.2) [23]	18 (14.0) [36]	11 (9.7) [21]	7 (6.8) [13]
AEs leading to study drug discontinuation	6 (4.6) [7]	4 (3.1) [4]	1 (0.9) [1]	3 (2.9) [4]
Treatment-related AEs leading to study drug discontinuation	—	—	1 (0.9) [1]	1 (1.0) [1]
AEs leading to early study termination	5 (3.8) [5]	4 (3.1) [4]	1 (0.9) [1]	3 (2.9) [4]
AEs leading to death	0	0	0	0
**Treatment-related AEs reported by ≥2% of patients during the treatment period**				
Patients with any related event	37 (28.5) [118]	49 (38.0) [102]	26 (23.0) [92]	18 (17.5) [38]
Nervous system disorders	9 (6.9) [22]	17 (13.2) [22]	5 (4.4) [9]	3 (2.9) [4]
Dizziness	4 (3.1) [11]	9 (7.0) [10]	3 (2.7) [6]	1 (1.0) [1]
Somnolence	2 (1.5) [3]	3 (2.3) [3]	—	—
Gastrointestinal disorders	4 (3.1) [5]	12 (9.3) [16]	—	—
Constipation	1 (0.8) [1]	4 (3.1) [4]	—	—
Diarrhoea	2 (1.5) [2]	3 (2.3) [3]	—	—
Nausea	0	3 (2.3) [3]	—	—
Metabolism and nutrition disorders	16 (12.3) [21]	11 (8.5) [16]	11 (9.7) [18]	11 (10.7) [19]
Hyperkalaemia	13 (10.0) [15]	11 (8.5) [15]	10 (8.8) [17]	11 (10.7) [19]
Cardiac disorders	9 (6.9) [42]	8 (6.2) [11]	6 (5.3) [49]	1 (1.0) [1]
Palpitations	4 (3.1) [5]	3 (2.3) [3]	3 (2.7) [5]	0
Tachycardia	5 (3.8) [36]	3 (2.3) [6]	5 (4.4) [44]	1 (1.0) [1]
Psychiatric disorders	4 (3.1) [7]	6 (4.7) [14]	—	—
Insomnia	3 (2.3) [3]	3 (2.3) [7]	—	—
Skin and subcutaneous disorders	1 (0.8) [1]	5 (3.9) [8]	—	—
Rash	1 (0.8) [1]	3 (2.3) [6]	—	—
Vascular disorders	2 (1.5) [3]	4 (3.1) [5]	—	—
Hypotension	2 (1.5) [3]	4 (3.1) [5]	—	—

SAE, serious AE.

## DISCUSSION

This Phase 3 study met its primary efficacy endpoint: patients treated with difelikefalin showed significantly greater WI-NRS improvement from baseline at Week 4 versus placebo, confirming the efficacy of difelikefalin in this Chinese population. Efficacy was shown to be robust, supported by two sensitivity analyses. Although this study is limited to a Chinese population, the primary endpoint result (Fig. 2) was comparable to the results of previous studies conducted outside of China [21, 22] (Supplimentary Fig. 1). In a Phase 3 study in Japan, the LS mean change from baseline in the WI-NRS score (95% CI) at Week 4 (the primary efficacy endpoint) was greater with difelikefalin versus placebo, with a significant difference between the groups: −0.97 (−1.52, −0.42), *P* < .001 [22]. A greater improvement with difelikefalin versus placebo was also seen in a pooled analysis of the pivotal Phase 3 KALM-1 and KALM-2 studies, in which the difference between the groups was −0.8 (95% CI −1.1, −0.5), *P* < .001 [21] (Supplementary Table 6).

This study, similar to previous studies, detected greater WI-NRS improvement with difelikefalin versus placebo as early as Week 2, which continued throughout the double-blind period [21, 22]. The ability to consistently detect robust treatment effects as early as Week 4 supports shorter double-blind periods in this therapeutic area, such as the 6-week double-blind period used in the Phase 3 study of difelikefalin in Japan [22].

A ≥3-point reduction in WI-NRS score represents a clinically relevant reduction in pruritus intensity in patients with moderate-to-severe CKD-aP [23, 24]. The percentage of participants achieving a ≥3-point improvement in the weekly mean of WI-NRS scores at Week 12 of the double-blind period in the current study was comparable with that observed in the KALM-1 and KALM-2 studies (LS means estimate of 50.9% and 53.4%, respectively, outcome defined as primary endpoint in these studies) [21] (Supplementary Fig. 2C). Thus, all WI-NRS endpoints demonstrated consistency across populations.

CKD-aP can markedly impair the HRQoL of patients receiving HD [1–5]. Greater improvements in 5-D itch and Skindex-10 in terms of total scores and percentage of patients with clinically relevant changes were observed with difelikefalin compared with placebo during the double-blind period of this study. After switching treatment from placebo to difelikefalin at the start of the OLE, patients experienced improvements in 5-D itch and Skindex-10 total scores similar to those of patients continuously treated with difelikefalin. In addition, the estimated percentage of patients with a clinically relevant improvement in 5-D itch and Skindex-10 increased by ∼20 percentage points from double-blind Week 12 to open-label Week 4. From Weeks 4 to 14 of the open-label period, most patients in both groups maintained clinically relevant improvements in HRQoL measures (estimated percentage of ≥62% for 5-D itch and ≥56% for Skindex-10). Throughout the study, patients on difelikefalin continued to experience improvements in HRQoL. This demonstrates the benefit and importance of continuous treatment with difelikefalin over a long period.

Treatment with difelikefalin considerably increased the percentage of responders (those reporting that their pruritus had ‘very much improved’ or ‘much improved’) in the PGI-C at the end of the 12-week double-blind treatment period indicating that patients perceived meaningful improvement of symptoms with treatment.

Difelikefalin was generally well-tolerated in this study. Safety results over 26 weeks of treatment were generally consistent with the known safety profile of difelikefalin [17]. However, a higher reporting frequency was observed for some AEs compared with other studies.

The most frequently reported treatment-related AE in this study was hyperkalaemia. Hyperkalaemia is prevalent in patients with CKD, particularly at more advanced stages of CKD [25, 26]. The similar incidence rates of hyperkalaemia between difelikefalin and placebo groups reported in the current study indicate that difelikefalin does not increase the risk of hyperkalaemia.

Muscle spasms were more frequently observed in the difelikefalin group than the placebo group. However, no treatment-related muscle spasms were reported during the double-blind period, and only one related event occurred during the open-label period. Notably, individuals receiving dialysis are predisposed to muscle-related symptoms due to intradialytic hypotension, electrolyte imbalance, and muscle waste [27].

Hypotension and dialysis hypotension were also frequently reported and occurred more often in the difelikefalin group than in the placebo group. While the activation of κ-opioid receptors by difelikefalin could potentially lead to vasodilation and, therefore, contribute to hypotension, clinical and preclinical studies indicated that most hypotension episodes following difelikefalin treatment were transient, and not determined to be directly related to treatment [28]. In line with this, most hypotension episodes experienced during the study were transient and resolved on the same day. With the exception of one serious and severe treatment-related AE of hypotension reported in one patient (0.8%) in the difelikefalin group, all AEs of hypotension or dialysis hypotension mild or moderate in intensity. A systematic review indicates that the prevalence of intradialytic hypotension in patients on HD in China is approximately 35%, with an incidence of approximately 7% and an upward trend [29]. The reporting rates of hypotension, dialysis hypotension, and muscle spasms in this study may, in part, reflect the high prevalence of intradialytic hypotension in China [29, 30]. The observed differences between treatment groups underscore the need for further investigation and continued monitoring.

In a Phase 3 study of nalfurafine in Chinese patients on HD with refractory CKD-aP, insomnia, and irritability were observed as common adverse drug reactions (22.8% and 5.3%, respectively, in the group receiving the dose of 5 µg), which might be mediated by the CNS [31]. By contrast, in the double-blind period of the current study, insomnia was reported as an AE related to the treatment in 2.3% of patients in either treatment group.

It is noteworthy that most (88.8%) patients in this study were not using an anti-pruritic medication in the week before randomization, despite >50% of patients experiencing severe CKD-aP. While it is generally known that CKD-aP is undertreated [7], the large percentage of patients not receiving previous anti-pruritic medications underlines the need for increasing awareness of CKD-aP and effective treatments for this condition in China.

A limitation of this study is the lack of an active comparator. For the pivotal Phase 3 KALM studies, no other treatments specifically for CKD-aP were available in the countries where these studies were conducted. Nalfurafine hydrochloride is available in China; however, it was not yet approved at the time this study was initiated, and consequently, a comparison with nalfurafine hydrochloride was not within the scope of this study. Other limitations include the smaller sample size and the shorter duration of the OLE compared to the KALM studies.

The results of this study showed a favourable benefit–risk profile supporting difelikefalin use in Chinese patients receiving HD, while also highlighting the need for better awareness of CKD-aP in this population. The treatment effect of difelikefalin in this study was comparable with the results of Phase 3 studies conducted in different regions of the world, despite differences in study design, patient background, dialysis practice, and available treatments. This study strengthens the evidence for the efficacy of difelikefalin in reducing pruritus intensity and improving the HRQoL in patients receiving HD across different ethnic populations, while showing an acceptable safety profile.

## Supplementary Material

sfag031_Supplemental_File

## Data Availability

Data are available at https://clinicaltrials.gov/study/NCT05885737?tab=results.
