# Supplementary material for: Difelikefalin in Chinese patients with chronic kidney disease-associated pruritus
Source: Clin Kidney J. 2026 Feb 6;19(4):sfag031. doi: 10.1093/ckj/sfag031 (PMC13053084; doi:10.1093/ckj/sfag031)
Supplement: sfag031_Supplemental_File [file sfag031_supplemental_file.docx]

# SUPPLEMENTARY MATERIAL:

### **1. Inclusion and Exclusion Criteria**

*1.1 Inclusion Criteria*

Subjects were eligible for the double-blind period of the study if they met all the following inclusion criteria:

1. Subject (or legally accepted representative) provided written informed consent. Written informed consent was to be provided before any study-specific procedures were performed, including screening procedures.
2. Chinese subjects aged ≥18 to 85 years (inclusive) at the time of consent.
3. Able to communicate clearly with the Investigator and staff, able to understand the study procedures, and able and willing to comply with the study requirements, including providing written responses to questionnaires.
4. Subjects with chronic kidney disease (CKD) on haemodialysis (HD) 3 times weekly for ≥12 weeks prior to the informed consent procedure (including the date of informed consent) who could continue HD without changing its frequency or method.
   - Note 1: Any temporary change in dialysis frequency or method associated with travel or admission to other hospitals with no changes in treatment strategies was acceptable, from the date of informed consent until the end of the follow-up period. Subjects routinely on four dialyses a week were not eligible.
   - Note 2: Subjects receiving in-home HD could participate as long as they had switched to in-centre HD at least 2 weeks prior to screening and planned to remain on in-centre HD for the duration of the study.
   - Note 3: Subjects receiving alternate dialysis modalities such as nocturnal dialysis were not eligible.
5. If female, was not pregnant, or nursing.
6. If female:
   1. Was surgically sterile; or
   2. Had been amenorrhoeic for at least 1 year and was over the age of 55 years; or
   3. Had a negative serum pregnancy test within 7 days before first dose of investigational product and agreed to use acceptable contraceptive measures (e.g. hormonal contraceptives, barrier with spermicide, intrauterine device, vasectomised partner, or abstinence) from the time of informed consent until 7 days after the last dose of investigational product. Note: If the result from serum pregnancy testing at screening was indeterminate due to possible human chorionic gonadotropin elevation secondary to end-stage renal disease (ESRD) unrelated to pregnancy, a serum pregnancy re-test could be repeated prior to treatment Day 1 to establish if a negative result could be confirmed.
7. If male, agreed not to donate sperm after the first dose of investigational product administration until 7 days after the last dose of investigational product, and agreed to use a condom with spermicide or abstain from heterosexual intercourse during the study until 7 days after the last dose of investigational product. Note: No restrictions were required for a vasectomised male, provided his vasectomy was performed ≥4 months prior to screening.
8. Subjects whose Numerical Rating Scale (NRS) score in the 7-day run-in period (7 days including the score recorded on the start day of treatment) met both of the below criteria:
   1. NRS scores had been recorded for at least 4 days through a 7-day run-in period.
   2. The mean value of the recorded scores was ≥5.0 (moderate-to-severe pruritus).
9. Subjects with a prescription dry body weight between 40 and 100 kg, inclusive.
10. Over the last 3 months prior to screening, had at least 1 of the following:
    1. At least two single-pool Kt/V measurements ≥1.2 on different dialysis days [Kt/V = (dialyzer clearance of urea × dialysis time) / volume of distribution of urea]
    2. At least two urea reduction ratio measurements ≥65% on different dialysis days
    3. One single-pool Kt/V measurement ≥1.2 and one urea reduction ratio measurement ≥65% on different dialysis days

Additionally, subjects were eligible for the optional open-label extension if they met all of the following inclusion criteria at the end of Week 12 of the double-blind period:

1. Subjects not withdrawn during the double-blind period.
2. Subjects receiving at least 30 doses of the planned 36 doses of investigational product during the double-blind period.
3. Subject had a prescription dry body weight ≥40 kg.
4. Subject continued to meet inclusion criteria 1 through 7 of the double-blind period.
5. Subjects did not have any safety or other reasons, which in the opinion of the Investigator, should have excluded them from entering the open-label extension period.

*1.2 Exclusion Criteria*

Subjects were excluded from participation if any of the following exclusion criteria were met:

1. Known non-compliance with dialysis treatment that in the opinion of the Investigator would impede completion or validity of the study.

2. Planned or anticipated to receive a kidney transplant during the study. Note: Being listed on a kidney transplant list was not an exclusion criterion.

3. Subjects with itching caused by conditions other than chronic renal failure or complications of chronic renal failure, which could affect the efficacy evaluation in the opinion of the Investigator (e.g. atopic dermatitis, chronic urticaria). Note: Subjects whose pruritus was attributed to ESRD complications, such as hyperparathyroidism, hyperphosphataemia, anaemia, or the dialysis procedure, or prescription, could be enrolled.

4. Had localised itch restricted to the palms of the hands.

5. Had pruritus only during the dialysis session (by subject report).

6. Subjects with severe hepatic impairment (Child-Pugh Class C) or concurrent hepatic cirrhosis.

7. Subject was receiving ongoing ultraviolet B treatment and anticipated receiving such treatment during the study.

8. Subjects who previously were enrolled in any clinical study of difelikefalin and received at least one dose of difelikefalin.

9. Significant systolic or diastolic heart failure (e.g. New York Heart Association Class IV congestive heart failure).

10. Subjects with concurrent malignancy except excised basal cell or squamous cell carcinoma of the skin, or carcinoma *in situ* that had been excised or resected completely.

11. Known or suspected history of alcohol, narcotic, or other drug abuse, or substance dependence within 12 months prior to screening.

12. Severe mental illness or cognitive impairment (e.g. dementia) or other concurrent mental disorder that, in the opinion of the Investigator, would compromise the validity of study measurements.

13. Any other relevant acute or chronic medical or neuropsychiatric condition within 3 months prior to screening (e.g. diagnosis of encephalopathy, coma, delirium).

14. New or change of treatment received for itch including antihistamines and corticosteroids (oral, intravenous, or topical) within 14 days prior to screening.

15. New or change of prescription for opioids, gabapentin, or pregabalin within 14 days prior to screening.

16. Subject was receiving prohibited medication (e.g. nalfurafine hydrochloride, opioid antagonists).

17. Subjects who received treatment with any investigational product or study device in a clinical study (including clinical studies of medical devices or cellular and tissue-based products) within 30 days prior to the informed consent procedure, or who were planning to participate in another clinical study before the end of the follow-up period of this study.

### **2. Statistical Analyses**

### 2.1 Sample Size Calculation

Based on the results observed at the end of Week 4 in previous studies, a mean difference of −0.9 with a common standard deviation of 2.1 was assumed between difelikefalin and placebo groups regarding the primary endpoint.

Assuming a 2-sided significance level of 5% and a statistical power of 90%, 116 subjects per group were required to detect a difference of −0.9 (with a common standard deviation of 2.1) between the difelikefalin and the placebo groups using a 2‑sample t-test.

Assuming a 10% dropout rate, 258 subjects in total were needed to be enrolled in the double-blind study period, that is, 129 subjects per treatment group.

*2.2 Primary Endpoint Analysis*

The full analysis set – defined as all patients who were randomised, received ≥1 dose of investigational product, and had a baseline Worst Itching Intensity Numerical Rating Scale (WI-NRS) score – was used to analyse the primary efficacy endpoint. Significance level was set at an α of 0.05 (2-sided). The treatment groups were compared using the least squares (LS) means of change from baseline to Week 4 in the weekly mean of the WI-NRS score estimated using a mixed-effect model for repeated measures (MMRM) that included treatment, week, and treatment-by-week interaction as fixed effects. In this model, baseline weekly mean WI‑NRS score, use of prior anti-pruritic medication, and presence of specific medical conditions (i.e. history of fall or fracture [related to fall], confusional state or mental status change or altered mental status or disorientation, gait disturbance or movement disorder) were used as covariates. Missing mean WI NRS scores were not imputed, assuming they were missing at random as per the MMRM.

Sensitivity analyses were performed for the primary endpoint to test the robustness of the assumptions used in the primary analysis with respect to missing data.

*2.3 Secondary Efficacy Endpoint Analyses*

For the analysis of percentage of patients with an improvement in WI-NRS score of ≥3 or ≥4 points, all available WI-NRS scores were included in the analysis. Missing WI-NRS scores were imputed using a multiple imputation, missing at random approach. After imputation of missing data, the percentage of patients who improved from baseline with respect to the WI-NRS score was calculated for each imputed dataset. Results of the logistic regression on the multiply imputed data sets were summarised using Rubin’s rule. The observed number and percentage of patients achieving ≥3-point and ≥4-point improvement, respectively, among the non-imputed data were recorded along with the imputed data logistic regression model-based estimates of the percentages of responders with their 2-sided 95% confidence interval (CI).

The total scores and domains of the 5-D itch scale and Skindex-10 were analysed using the same MMRM method as the primary efficacy analysis. The logistic regression model-based estimates of the percentages of patients achieving ≥5-point improvements in 5-D itch scale and ≥15-point improvements in Skindex-10 were reported along with their 2-sided 95% CIs.

All Patient Global Impression of Change (PGI-C) data were included in the analysis. Missing PGI-C data were not imputed. The number and percentage of patients for each response (‘Very Much Improved’, ‘Much Improved’, ‘Minimally Improved’, ‘No Change’, ‘Minimally Worse’, ‘Much Worse’, and ‘Very Much Worse’) were reported, as well as the number and percentage of responders, defined as patients who responded, ‘Very Much Improved’ or ‘Much Improved’.

*2.4 Safety Endpoint Analyses*

Safety data are presented descriptively.

**4. Supplementary Tables and Figures

Supplementary Table 1.** Sensitivity analyses: MMRM analysis of change from baseline to Week 4 of the double-blind period in the weekly mean of the daily 24-hour WI-NRS score – multiple imputation

| **Visit**  **Statistic** | **Placebo (*n* = 130)** | | | **Difelikefalin (*n* = 129)** | |
| --- | --- | --- | --- | --- | --- |
|  | **Value** | **Change from baseline** | | **Value** | **Change from baseline** |
| **Baseline** |  |  | |  |  |
| *n* (missing) | 130 (0) |  | | 129 (0) |  |
| Mean (SD) | 7.00 (1.223) |  | | 7.35 (1.291) |  |
| Median | 7.00 |  | | 7.38 |  |
| Min, Max | 5.0, 10.0 |  | | 5.0, 10.0 |  |
| **Week 4** (Observed) |  |  | |  |  |
| *n* (missing) | 126 (4) | 126 (4) | | 119 (10) | 119 (10) |
| Mean (SD) | 5.77 (1.679) | −1.22 (1.448) | | 5.45 (2.090) | −1.91 (1.907) |
| Median | 5.93 | −1.13 | | 5.71 | −1.43 |
| Min, Max | 1.7, 10.0 | −5.3, 1.7 | | 0.0, 10.0 | −9.0, 1.8 |
| **Sensitivity analysis #1^a^** | | | | | |
| LS mean (SE) |  | −1.28 (0.190) | |  | −2.05 (0.194) |
| 95% CI |  | −1.65, −0.91 | |  | −2.44, −1.67 |
| LS mean difference (SE) (difelikefalin minus placebo) | | | | | −0.78 (0.219) |
| 95% CI |  |  | |  | −1.21, −0.35 |
| *P*-value |  |  | |  | 0.0004 |
| **Sensitivity analysis #2^b^** | | | | | |
| LS mean (SE) |  | | −1.28 (0.190) |  | −2.04 (0.195) |
| 95% CI |  | | −1.65, −0.91 |  | −2.42, −1.66 |
| LS mean difference (SE) (difelikefalin minus placebo) | | | | | −0.76 (0.217) |
| 95% CI |  | |  |  | −1.18, −0.33 |
| *P*-value |  | |  |  | 0.0005 |

CI, confidence interval; LS, least squares; MAR, missing at random; MI, multiple imputation; MMRM, mixed model for repeated measures; MNAR, missing not at random; SD, standard deviation; SE, standard error; WI-NRS, Worst Itching Intensity Numerical Rating Scale.
Baseline WI-NRS score is calculated using all available non-missing scores collected on or before day of randomisation, and, prior to date/time of first treatment. The MMRM includes use of prior anti-pruritic medication (yes/no), presence of specific medical conditions at baseline (yes/no), treatment, visit, and treatment-by-visit-interaction as fixed categorical effects, and baseline WI-NRS score as fixed continuous effects. Variance-Covariance structure = Unstructured. Repeated measures up to Week 12 are included in the model.
^a^Sensitivity analysis #1 used MI MAR assumption. Missing values were imputed using MI under MAR missing data assumption.
^b^Sensitivity analysis #2 used placebo MI with MNAR assumption. Missing values were imputed using placebo MI under MNAR missing data assumption.

**Supplementary Table 2.** Summary of change from baseline in 5-D itch total score at Weeks 4, 8 and 12 of the double-blind period

| **Visit**  **Statistic** | **Placebo (*n* = 130)** | | **Difelikefalin (*n* = 129)** | |
| --- | --- | --- | --- | --- |
|  | **Value** | **Change from baseline** | **Value** | **Change from baseline** |
| **Baseline** |  |  |  |  |
| *n* (missing) | 130 (0) |  | 129 (0) |  |
| Mean (SD) | 15.5 (3.29) |  | 15.9 (3.36) |  |
| Median | 15.0 |  | 16.0 |  |
| Min, Max | 7.0, 24.0 |  | 10.0, 25.0 |  |
|  |  |  |  |  |
| **Week 4** (Observed) |  |  |  |  |
| *n* (missing) | 125 (5) | 125 (5) | 119 (10) | 119 (10) |
| Mean (SD) | 12.9 (3.05) | −2.6 (2.90) | 12.3 (3.14) | −3.7 (3.36) |
| Median | 13.0 | −3.0 | 12.0 | −4.0 |
| Min, Max | 7, 22 | −12, 5 | 5, 23 | −13, 4 |
| LS mean (SE) |  | −2.5 (0.36) |  | −3.3 (0.37) |
| 95% CI |  | −3.2, −1.8 |  | −4.1, −2.6 |
| LS mean difference (SE) (difelikefalin minus placebo) | | | | −0.8 (0.34) |
| 95% CI |  |  |  | −1.5, −0.2 |
| *P*-value |  |  |  | 0.0139 |
|  |  |  |  |  |
| **Week 8** (Observed) |  |  |  |  |
| *n* (missing) | 121 (9) | 121 (9) | 114 (15) | 114 (15) |
| Mean (SD) | 12.7 (3.33) | −2.9 (3.09) | 11.6 (3.21) | −4.3 (3.65) |
| Median | 12.0 | −3.0 | 11.5 | −4.0 |
| Min, Max | 6, 21 | −11, 3 | 5, 20 | −15, 4 |
| LS mean (SE) |  | −2.8 (0.37) |  | −3.9 (0.38) |
| 95% CI |  | −3.5, −2.0 |  | −4.7, −3.2 |
| LS mean difference (SE) (difelikefalin minus placebo) | | | | −1.2 (0.37) |
| 95% CI |  |  |  | −1.9, −0.4 |
| *P*-value |  |  |  | 0.0018 |
|  |  |  |  |  |
| **Week 12** (Observed) |  |  |  |  |
| *n* (missing) | 119 (11) | 119 (11) | 111 (18) | 111 (18) |
| Mean (SD) | 11.8 (3.48) | −3.7 (3.11) | 11.2 (3.45) | −4.7 (3.90) |
| Median | 11.0 | −4.0 | 11.0 | −4.0 |
| Min, Max | 5.0, 24.0 | −13.0, 3.0 | 5.0, 23.0 | −14.0, 7.0 |
| LS mean (SE) |  | −3.6 (0.39) |  | −4.3 (0.40) |
| 95% CI |  | −4.4, −2.8 |  | −5.1, −3.5 |
| LS mean difference (SE) (difelikefalin minus placebo) | | | | −0.7 (0.40) |
| 95% CI |  |  |  | −1.5, 0.1 |
| *P*-value |  |  |  | 0.0887 |

CI, confidence interval; LS, least squares; n, number of subjects; SD, standard deviation; SE, standard error.
Baseline is defined as the last available value (scheduled or unscheduled) before or on the same day as randomisation or if missing as any values during Week 1 (Day ≤7). The mixed model for repeated measures includes use of prior anti-pruritic medication (yes/no), presence of specific medical conditions at baseline (yes/no), treatment, visit, and treatment-by-visit-interaction as fixed categorical effects and baseline 5-D itch score (total score or each domain score) as fixed continuous effects. Variance-covariance structure is unstructured. Repeated measures up to Week 12 are included in the model.

**Supplementary Table 3.** Summary of change from baseline in Skindex-10 total score at Weeks 4, 8 and 12 of the double-blind period

| **Visit**  **Statistic** | **Placebo (*n* = 130)** | | **Difelikefalin (*n* = 129)** | |
| --- | --- | --- | --- | --- |
|  | **Value** | **Change from baseline** | **Value** | **Change from baseline** |
| **Baseline** |  |  |  |  |
| *n* (missing) | 130 (0) |  | 129 (0) |  |
| Mean (SD) | 28.8 (15.16) |  | 29.6 (15.77) |  |
| Median | 28.0 |  | 29.0 |  |
| Min, Max | 0.0, 60.0 |  | 5.0, 60.0 |  |
|  |  |  |  |  |
| **Week 4** (Observed) |  |  |  |  |
| *n* (missing) | 124 (6) | 124 (6) | 120 (9) | 120 (9) |
| Mean (SD) | 21.5 (14.47) | −7.1 (11.77) | 18.0 (13.63) | −11.6 (12.75) |
| Median | 18.0 | −5.5 | 14.5 | −10.0 |
| Min, Max | 0, 60 | −40, 27 | 0, 60 | −49, 18 |
| LS mean (SE) |  | −5.1 (1.43) |  | −9.1 (1.46) |
| 95% CI |  | −8.0, −2.3 |  | −12.0, −6.3) |
| LS mean difference (SE) (difelikefalin minus placebo) | | | | −4.0 (1.36) |
| 95% CI |  |  |  | −6.7, −1.3 |
| *P*-value |  |  |  | 0.0038 |
|  |  |  |  |  |
| **Week 8** (Observed) |  |  |  |  |
| n (missing) | 121 (9) | 121 (9) | 115 (14) | 115 (14) |
| Mean (SD) | 19.2 (15.25) | −9.3 (12.65) | 15.7 (12.73) | −13.9 (14.28) |
| Median | 14.0 | −9.0 | 13.0 | −13.0 |
| Min, Max | 0, 60 | −46, 20 | 0, 59 | −56, 23 |
| LS mean (SE) |  | −7.4 (1.48) |  | −11.3 (1.51) |
| 95% CI |  | −10.3, −4.5 |  | −14.3, −8.3 |
| LS mean difference (SE) (difelikefalin minus placebo) | | | | −3.9 (1.46) |
| 95% CI |  |  |  | −6.8, −1.0 |
| *P*-value |  |  |  | 0.0085 |
|  |  |  |  |  |
| **Week 12** (Observed) |  |  |  |  |
| *n* (missing) | 119 (11) | 119 (11) | 111 (18) | 111 (18) |
| Mean (SD) | 17.1 (15.21) | −11.6 (13.15) | 16.3 (13.91) | −13.0 (15.75) |
| Median | 12.0 | −9.0 | 14.0 | −13.0 |
| Min, Max | 0.0, 60.0 | −50.0, 18.0 | 0.0, 60.0 | −57, 27 |
| LS mean (SE) |  | −9.5 (1.55) |  | −10.5 (1.59) |
| 95% CI |  | −12.5, −6.4 |  | −13.6, −7.4 |
| LS mean difference (SE) (difelikefalin minus placebo) | | | | −1.1 (1.61) |
| 95% CI |  |  |  | −4.2, 2.1 |
| *P*-value |  |  |  | 0.5119 |

CI, confidence interval; LS, least squares; n, number of subjects; SD, standard deviation; SE, standard error.
Baseline is defined as the last available value (scheduled or unscheduled) before or on the same day as randomisation or if missing as any values during Week 1 (Day ≤7). The mixed model for repeated measures includes use of prior anti-pruritic medication (yes/no), presence of specific medical conditions at baseline (yes/no), treatment, visit, and treatment-by-visit-interaction as fixed categorical effects and baseline Skindex-10 score (total score or each domain score) as fixed continuous effects. Variance-covariance structure is unstructured. Repeated measures up to Week 12 are included in the model.

**Supplementary Table 4.** Summary of Patient Global Impression of Change at the end of Week 12 of the double-blind period

|  | | **Placebo**  **(*n* = 130)** | **Difelikefalin**  **(*n* = 129)** |
| --- | --- | --- | --- |
| *n* (missing) | | 119 (11) | 110 (19) |
| PGI-C response, *n* (%) | Very much improved | 14 (11.8) | 20 (18.2) |
|  | Much improved | 29 (24.4) | 46 (41.8) |
|  | Minimally improved | 49 (41.2) | 22 (20.0) |
|  | No change | 24 (20.2) | 18 (16.4) |
|  | Minimally worse | 2 (1.7) | 2 (1.8) |
|  | Very much worse | 1 (0.8) | 2 (1.8) |
| Percentage of responders^a^ (95% CI) | | 36.1 (27.5, 45.4) | 60.0 (50.2, 69.2) |
| Odds ratio (95% CI)^b^ | | 2.65 (1.49, 4.62) | |
| P-value^b^ | | 0.0004 | |

CI, confidence interval; PGI-C, Patient Global Impression of Change.
Percentages are based on the number of patients with non-missing assessment.
^a^Responders are those with responses of ‘Very Much Improved’ or ‘Much Improved’. CIs for frequencies are calculated as exact Clopper-Pearson 95% CIs.
^b^Odds ratio and its exact CI are based on the Mantel-Haenszel estimate stratified by use of anti-pruritic medication during the week prior to randomisation and the presence of specific medical conditions at baseline and taking the placebo treatment group as a reference. *P*-value is from the stratified Cochran-Mantel-Haenszel test.

**Supplementary Table 5.** Adverse events reported by ≥2% of patients in any treatment group during the whole study

| **System Organ Class**  **Preferred Term** | **Double-blind treatment period** | | **Open-label extension period** | |
| --- | --- | --- | --- | --- |
|  | **Placebo**  **(*n* = 130)**  ***n* (%) [E]** | **Difelikefalin**  **(*n* = 129)**  ***n* (%) [E]** | **Placebo/ difelikefalin**  **(*n* = 113)**  ***n* (%) [E]** | **Difelikefalin/ difelikefalin**  **(*n* = 103)**  ***n* (%) [E]** |
| Patients with any event | 92 (70.8) [383] | 106 (82.2) [501] | 90 (79.6) [466] | 82 (79.6) [407] |
| Vascular disorders | 23 (17.7) [58] | 45 (34.9) [115] | 31 (27.4) [105] | 26 (25.2) [83] |
| Hypotension | 8 (6.2) [11] | 21 (16.3) [30] | 16 (14.2) [29] | 12 (11.7) [13] |
| Dialysis hypotension | 9 (6.9) [26] | 19 (14.7) [71] | 16 (14.2) [63] | 13 (12.6) [55] |
| Hypertension | 5 (3.8) [7] | 5 (3.9) [5] | — | — |
| Dialysis-induced hypertension | 3 (2.3) [14] | 1 (0.8) [5] | — | — |
| Gastrointestinal disorders | 21 (16.2) [32] | 34 (26.4) [53] | 20 (17.7) [34] | 23 (22.3) [34] |
| Constipation | 4 (3.1) [4] | 9 (7.0) [11] | 1 (0.9) [3] | 4 (3.9) [4] |
| Diarrhoea | 6 (4.6) [6] | 6 (4.7) [6] | 3 (2.7) [3] | 7 (6.8) [7] |
| Nausea | 1 (0.8) [1] | 6 (4.7) [9] | — | — |
| Vomiting | 4 (3.1) [5] | 4 (3.1) [7] | — | — |
| Abdominal pain | — | — | 1 (0.9) [2] | 3 (2.9) [3] |
| Abdominal pain upper | 4 (3.1) [5] | 1 (0.8) [1] | 4 (3.5) [7] | 1 (1.0) [1] |
| Gastrointestinal disorder | 3 (2.3) [3] | 0 | — | — |
| Musculoskeletal and connective tissue disorders | 17 (13.1) [22] | 31 (24.0) [62] | 25 (22.1) [49] | 20 (19.4) [42] |
| Muscle spasms | 11 (8.5) [15] | 18 (14.0) [39] | 15 (13.3) [33] | 11 (10.7) [30] |
| Arthralgia | 1 (0.8) [1] | 4 (3.1) [4] | 5 (4.4) [5] | 3 (2.9) [4] |
| Back pain | — | — | 3 (2.7) [3] | 1 (1.0) [1] |
| Infections and infestations | 28 (21.5) [40] | 30 (23.3) [34] | 21 (18.6) [32] | 22 (21.4) [33] |
| Upper respiratory tract infection | 14 (10.8) [16] | 13 (10.1) [15] | 11 (9.7) [14] | 9 (8.7) [9] |
| Respiratory tract infection | 1 (0.8) [1] | 4 (3.1) [4] | 3 (2.7) [4] | 1 (1.0) [1] |
| Pneumonia | 6 (4.6) [8] | 2 (1.6) [2] | 6 (5.3) [6] | 4 (3.9) [4] |
| Skin infection | 3 (2.3) [5] | 1 (0.8) [1] | — | — |
| Gingivitis | 3 (2.3) [3] | 0 | — | — |
| Metabolism and nutrition disorders | 29 (22.3) [41] | 30 (23.3) [43] | 33 (29.2) [64] | 32 (31.1) [61] |
| Hyperkalaemia | 22 (16.9) [26] | 21 (16.3) [27] | 21 (18.6) [34] | 20 (19.4) [32] |
| Hypokalaemia | — | — | 2 (1.8) [2] | 4 (3.9) [4] |
| Hypercalcaemia | — | — | 6 (5.3) [6] | 5 (4.9) [5] |
| Hypocalcaemia | 2 (1.5) [3] | 3 (2.3) [3] | 2 (1.8) [2] | 7 (6.8) [7] |
| Hypoglycaemia | — | — | 5 (4.4) [5] | 4 (3.9) [4] |
| Nervous system disorders | 17 (13.1) [34] | 29 (22.5) [36] | 11 (9.7) [18] | 18 (17.5) [23] |
| Dizziness | 8 (6.2) [16] | 17 (13.2) [19] | 6 (5.3) [10] | 6 (5.8) [7] |
| Restless legs syndrome | 1 (0.8) [1] | 3 (2.3) [4] | 0 | 3 (2.9) [3] |
| Somnolence | 2 (1.5) [3] | 3 (2.3) [3] | — | — |
| Headache | 3 (2.3) [7] | 2 (1.6) [2] | 1 (0.9) [2] | 3 (2.9) [3] |
| Hypoaesthesia | — | — | 1 (0.9) [1] | 3 (2.9) [4] |
| General disorders and administration site conditions | 13 (10.0) [22] | 21 (16.3) [30] | 11 (9.7) [13] | 8 (7.8) [13] |
| Pyrexia | 4 (3.1) [4] | 7 (5.4) [7] | 4 (3.5) [4] | 1 (1.0) [1] |
| Chest discomfort | 6 (4.6) [6] | 6 (4.7) [8] | 2 (1.8) [2] | 3 (2.9) [5] |
| Fatigue | 0 | 6 (4.7) [9] | — | — |
| Cardiac disorders | 14 (10.8) [59] | 18 (14.0) [35] | 22 (19.5) [77] | 18 (17.5) [36] |
| Palpitations | 6 (4.6) [8] | 8 (6.2) [10] | 7 (6.2) [11] | 4 (3.9) [4] |
| Tachycardia | 6 (4.6) [37] | 6 (4.7) [16] | 9 (8.0) [48] | 3 (2.9) [4] |
| Cardiac failure | 4 (3.1) [7] | 0 | — | — |
| Coronary artery disease | — | — | 3 (2.7) [3] | 1 (1.0) [1] |
| Arteriosclerosis coronary artery | — | — | 0 | 3 (2.9) [3] |
| Injury, poisoning and procedural complications | 10 (7.7) [10] | 13 (10.1) [21] | — | — |
| Arteriovenous fistula site complication | 4 (3.1) [4] | 3 (2.3) [4] | — | — |
| Fall | 2 (1.5) [2] | 3 (2.3) [4] | — | — |
| Psychiatric disorders | 8 (6.2) [13] | 13 (10.1) [23] | 7 (6.2) [9] | 7 (6.8) [7] |
| Insomnia | 4 (3.1) [5] | 7 (5.4) [11] | — | — |
| Sleep disorder | — | — | 2 (1.8) [3] | 4 (3.9) [4] |
| Skin and subcutaneous tissue disorders | 5 (3.8) [5] | 10 (7.8) [13] | 6 (5.3) [6] | 4 (3.9) [4] |
| Dermatitis allergic | 0 | 3 (2.3) [3] | — | — |
| Rash | 2 (1.5) [2] | 3 (2.3) [6] | — | — |
| Hyperhidrosis | — | — | 3 (2.7) [3] | 0 |
| Respiratory, thoracic, and mediastinal disorders | 13 (10.0) [17] | 8 (6.2) [11] | 8 (7.1) [10] | 8 (7.8) [13] |
| Cough | 7 (5.4) [8] | 4 (3.1) [5] | 2 (1.8) [2] | 6 (5.8) [8] |
| Blood and lymphatic system disorders | — | — | 3 (2.7) [7] | 7 (6.8) [11] |
| Thrombocytopenia | — | — | 1 (0.9) [1] | 4 (3.9) [5] |

AE, adverse event; E, number of events; MedDRA, Medical Dictionary for Regulatory Activities; PT, preferred term; SOC, system organ class.
AEs are coded to SOC and PT using MedDRA, version 26.0. For each SOC and PT, patients are included only once, even if they experienced multiple events in that SOC or PT.

**Supplementary Table 6.** MMRM analysis of change from baseline to Week 4 of the double-blind period in the weekly mean of the daily 24-hour WI-NRS score in the pooled KALM-1 and KALM-2 dataset – no imputation

|  | **Difelikefalin**  **(*n* = 426)** | **Placebo (*n* = 425)** |
| --- | --- | --- |
| **Week 4** |  |  |
| Observed values |  |  |
| *N* | 390 | 403 |
| Mean (SD) | 4.9 (2.46) | 5.7 (2.25) |
| Median | 5.1 | 5.7 |
| Range (min, max) | (0, 10) | (0, 10) |
|  |  |  |
| Change from baseline |  |  |
| *N* | 390 | 403 |
| Mean (SD) | −2.3 (2.15) | −1.5 (1.99) |
| Median | −1.9 | −1.1 |
| Range (min, max) | (−10, 2) | (−9, 3) |
|  |  |  |
| LS mean | −2.2 | −1.4 |
| (SE) | (0.11) | (0.11) |
| 95% CI | −2.5, −2.0 | −1.6, −1.2 |
|  |  |  |
| Treatment difference  (difelikefalin – placebo) |  |  |
| LS mean |  | −0.8 |
| SE |  | 0.14 |
| 95% CI |  | −1.1, −0.5 |
| *P*-value |  | <0.001 |

CI, confidence interval; LS, least squares; MMRM, mixed model for repeated measures; SD, standard deviation; SE, standard error; WI-NRS, Worst Itching Intensity Numerical Rating Scale.
LS means, SEs, CIs, and *P*-values were based on an MMRM analysis with effects for treatment, visit, treatment-by-visit interaction, baseline score, use of anti-pruritic medication during the week prior to randomisation, and the presence of specific medical conditions. The model was fit using an unstructured covariance structure. A region/study combined variable was also included in the model for the pooled analysis.

**Supplementary Figure 1.** LS mean change from baseline in the weekly mean of the WI-NRS at Week 4 of the double-blind period of Phase 3 studies conducted in United States, global, Chinese, and Japanese populations

*
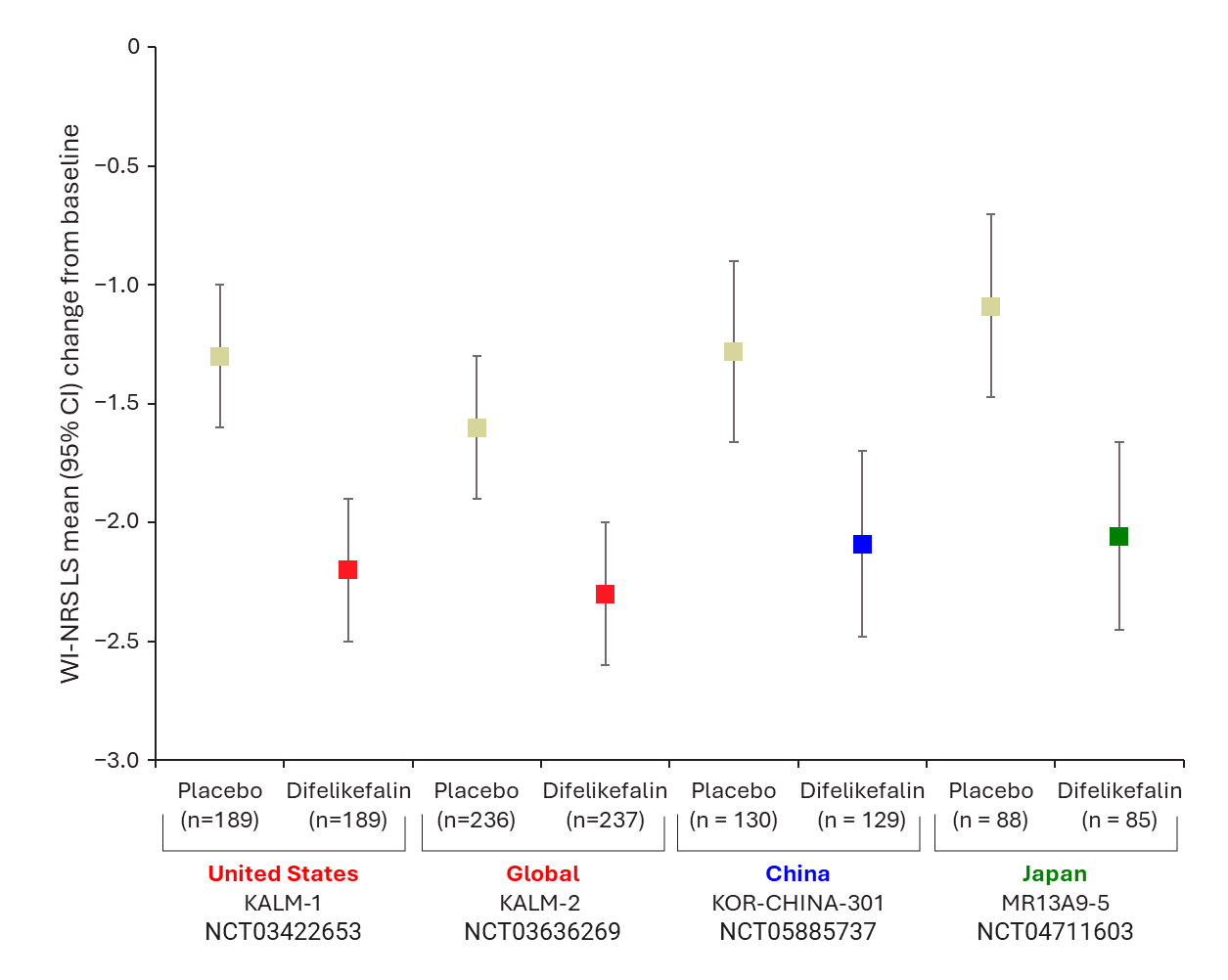
*

|  | **United States**  KALM-1  NCT03422653 | | **Global**  KALM-2  NCT03636269 | | **China**  KOR-CHINA-301 NCT05885737 | | **Japan**  MR13A9-5  NCT04711603 | |
| --- | --- | --- | --- | --- | --- | --- | --- | --- |
|  | **Placebo (*n* = 189)** | **Difelikefalin (*n* = 189)** | **Placebo (*n* = 236)** | **Difelikefalin (*n* = 237)** | **Placebo (*n* = 130)** | **Difelikefalin (*n* = 129)** | **Placebo  (*n* = 88)** | **Difelikefalin (*n* = 85)** |
| LS mean change from baseline at Week 4 | −1.3 | −2.2 | −1.6 | −2.3 | −1.3 | −2.1 | −1.1 | −2.1 |
| 95% CI | −1.6, −1.0 | −2.5, −1.9 | −1.9, −1.3 | −2.6, −2.0 | −1.7, −0.9 | −2.5, −1.7 | −1.5, −0.7 | −2.5, −1.7 |

CI, confidence interval; LS, least squares; WI-NRS, Worst Itching Intensity Numerical Rating Scale.

**Supplementary Figure 2.** LS means estimate of percentage of patients with ≥ 3-point improvement from baseline in the weekly mean of the WI-NRS by week in Phase 3 studies conducted in United States, global, and Chinese populations

| **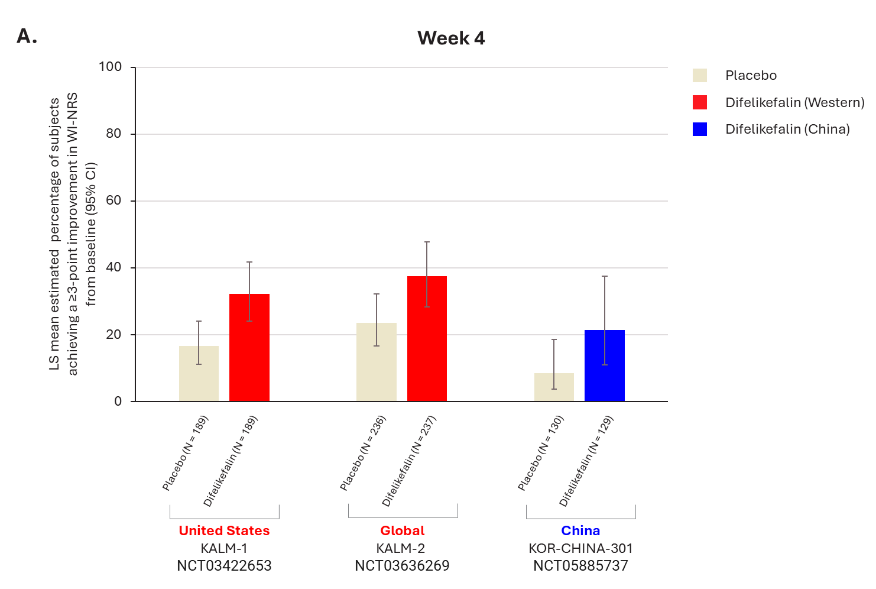** | |
| --- | --- |
| **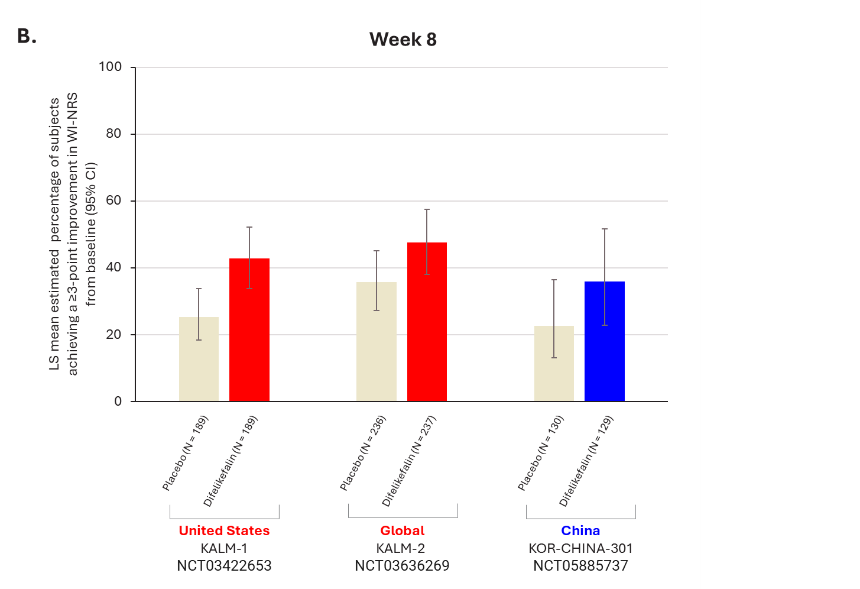** | **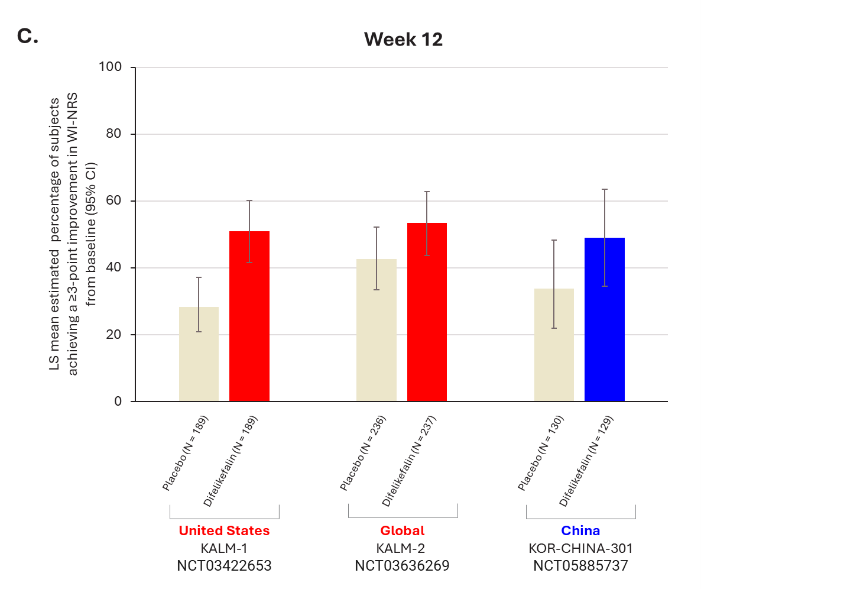** |

CI, confidence interval; LS, least squares; WI-NRS, Worst Itching Intensity Numerical Rating Scale.
